# Environmental Exposure to Cadmium and Lead Exacerbates Kidney Function in People with Diabetes

**DOI:** 10.3390/jox15060199

**Published:** 2025-12-01

**Authors:** Soisungwan Satarug, David A. Vesey, Tanaporn Khamphaya, Donrawee Waeyeng, Supabhorn Yimthiang

**Affiliations:** 1 Centre for Kidney Disease Research, The University of Queensland, Translational Research Institute, Woolloongabba, Brisbane, QLD 4102, Australia; david.vesey@health.qld.gov.au; 2 Department of Kidney and Transplant Services, Princess Alexandra Hospital, Brisbane, QLD 4102, Australia; 3 Occupational Health and Safety, School of Public Health, Walailak University, Nakhon Si Thammarat 80160, Thailand; tanaporn.kh@mail.wu.ac.th (T.K.); ksupapor@mail.wu.ac.th (S.Y.); 4 Environmental Health and Technology, School of Public Health, Walailak University, Nakhon Si Thammarat 80160, Thailand; donrawee.wae@wu.ac.th

**Keywords:** β_2_-microglobulin, cadmium, diabetes, eGFR, N-acetylglucosaminidase, lead

## Abstract

This study investigates the relationship between kidney function and exposure to low-level cadmium (Cd) and lead (Pb) in individuals with and without diabetes. Specifically, it tests the hypothesis that the nephrotoxicity of Cd and Pb reduces the tubular degradation of filtered proteins, namely β_2_-microglobulin (β_2_M). Data were obtained from a Thai cohort of 137 people, of which 65 were diagnosed with diabetes. Blood Cd, blood Pb, and urinary excretion of Cd (E_Cd_) were used as exposure indicators, while urinary N-acetylglucosaminidase (E_NAG_) and fractional tubular degradation of β_2_M (FrTD_β2M_) reflected kidney tubular cell injury and the function of tubular cells, respectively. Spearman’s rank correlation revealed that FrTD_β2M_ varied directly with the estimated glomerular filtration rate (eGFR; *r* = 0.434), and inversely with fasting plasma glucose (*r* = −0.215), E_Cd_ (*r* = −0.527), E_NAG_ (*r* = −0.536), and Cd/Pb exposure (*r* = −0.249). In a multiple regression model analysis adjusting for potential confounders, the association between FrTD_β2M_ and eGFR in those with diabetes was particularly strong (β = 0.476) compared to controls (β = 0.360), whereas an inverse association of FrTD_β2M_ and E_Cd_ (β = −0.295) was found only in those with diabetes, along with a positive association of E_NAG_ with E_Cd_ (R^2^ = 0.071). A mediation analysis has revealed that tubular injury (E_NAG_) mediated 26% of the FrTD_β2M_ decrease associated with Cd/Pb exposure. These findings suggested that tubular protein degradation pathways may be compromised under combined metabolic and environmental stressors, Cd, and Pb.

## 1. Introduction

Diabetes, defined as fasting plasma glucose (FPG) levels ≥ 126 mg/dL, is one of the chronic ailments with a high prevalence worldwide [1]. Among people with diabetes, hypertension and chronic kidney disease (CKD) are common comorbidities [2,3,4,5]. Notably, however, hypertension can be both a cause and a consequence of kidney damage [4,5,6]. The diagnosis of CKD is based on the presence of albuminuria for at least 3 months and/or a fall in the estimated glomerular filtration rate (eGFR) ≤ 60 mL/min/1.73 m^2^ [7,8,9]. Proteinuria is also a hallmark of kidney disease because it is a predictor of continued progressive eGFR decline to end-stage kidney disease [10,11,12,13,14], a condition that requires dialysis or a kidney transplant for survival. The associated healthcare costs are substantial.

Due to the widespread contamination of arable soils and staple foods, exposure to cadmium (Cd) and lead (Pb) is inevitable for most people [15,16]. This is reflected by several population studies that reported the association of CKD risk with exposure to the metals [17,18,19,20,21]. An association of Pb exposure with an increased CKD risk was found in non-smoking women, aged ≥ 50 years, who had diabetes and a body mass index (BMI) ≥ 25 kg/m^2^ [22]. In a cohort of Swedish women aged 64 years, an increased risk of albuminuria was found only in those with diabetes who had blood Cd within the top quartile [23]. A prospective cohort study from the Netherlands suggested that Cd exposure promoted eGFR deterioration in patients with diabetes [24].

The present study aimed to investigate the mechanisms of the nephrotoxicity of Cd and Pb in people with diabetes, emphasizing the connection between kidney tubular cell injury and the catabolism of filtered proteins, notably β_2_-microglobulin (β_2_M). We hypothesize that Cd/Pb exposure and/or diabetes (hyperglycemia) causes injury/damage to kidney tubular cells, which, in turn, diminishes the catabolism of filtered proteins, leading eventually to proteinuria and a declining eGFR.

Our hypothesis was constructed from our previous observations: a decrease in the fractional tubular degradation of β_2_M (FrTD_β2M_) due to diabetes [25] and increased risks of hyperglycemia, eGFR reduction, and albuminuria due to Cd/Pb exposure [26]. Studies by others have found that the risks of hypertension, kidney damage, and diabetes-related mortality may rise with an increment of plasma β_2_M levels [27,28,29]. Through the *SH2B3*-β_2_M axis of blood pressure control, β_2_M has been causally linked to hypertension development and kidney damage [30,31,32]. For these reasons, the present study has its focus on FrTD_β2M_.

## 2. Materials and Methods

### 2.1. Selection of Study Subjects

Data analyzed in the present study were from 137 people, enlisted from a pre-existing cohort of 186 adults (88 with diabetes and 88 without diabetes). As detailed previously [26], the enrolment criteria for participants in a pre-existing cohort included living at current addresses for at least 40 years and attending annual health checkups at the Pak Poon municipality health center, Nakhon Si Thammarat Province, Thailand. Non-resident persons and pregnant and breast-feeding women were excluded, as were those with records of ill-health status, such as heart disease, stroke, and cancer.

Participants were informed of study objectives and study protocols, along with potential risks and benefits. They gave written informed consent. Sociodemographic information, educational attainment, and occupation were collected using structured interview questionnaires, as were health status, family history of diabetes, use of dietary supplements, alcohol consumption, and smoking status. Special diets were not practiced in either group. Reported use of dietary supplements was 3% among controls and 7% in the diabetes group [33]. After individuals with missing data were excluded, a total of 137 individuals (65 with diabetes and 72 without diabetes) were analyzed in the present study. The % of hypertension in subjects with diabetes and without were 65 and 45, respectively.

### 2.2. Procurement and Storage of Blood and Urine Samples

Whole blood and urine samples were collected in the morning after an overnight fast at the Pak Poon health center. Urine samples were collected in metal-free polypropylene collection cups. For the glucose assay, blood samples were collected in heparinized tubes, using fluoride as an inhibitor of glycolysis. Ethylene diamine tetra acetic acid was an anticoagulant in collecting blood samples for Cd and Pb determination. All test tubes, bottles, and pipettes used in metal analysis were acid-washed and rinsed thoroughly with deionized water.

Blood and urine samples were kept on ice during transportation to the laboratory to prepare various aliquots of plasma and serum. The pH of urine aliquots was adjusted to >6 using 1 M NaOH to prevent the degradation of β_2_M in acidic conditions. Aliquots of urine, whole blood, serum, and plasma were stored at −80 °C for later analysis.

### 2.3. Quantification of Metals and Biomarkers of Kidney Effects

Urinary and serum concentrations of β_2_M were determined using the human beta-2 microglobulin/β_2_M ELISA pair set (Sino Biological Inc., Wayne, PA, USA). The lower limit of β_2_M detection was 3.13 pg/mL. The oxidase–peroxidase method (Glu Colorimetric Assay Kit, Elabscience, Catalog No: E-BC-K234-M, Houston, TX, USA) was used to determine plasma glucose concentrations [34]. The urinary NAG assay was based on colorimetry, using 4-nitrophenyl N-acetyl-β-D-glucosaminide as a substrate (Merck KGaA, Darmstadt, Germany). Jaffe’s alkaline picrate method was used to determine urinary and plasma creatinine concentrations [35]. All assays gave coefficients of variation (CVs) within acceptable clinical chemistry standards.

Graphite furnace atomic absorption spectrometry (GBC Scientific Equipment, Hampshire, IL, USA) was employed to determine Cd and Pb concentrations in urine and whole blood samples. To prepare samples for AAS, the deproteinization of blood samples with 5% HNO_3_ was used as described by Trzcinka-Ochocka et al. [36]. A standard with nine elements, As, Be, Cd, Cr (VI), Hg, Ni, Pb, Se, and Tl, was a certified reference material (Centipur^®^, Merck KGaA, Darmstadt, Germany), usable also as the instrumental calibrator. Duplicate samples were analyzed for consistency checks. Blank samples were included for contamination monitoring. Reference urine and whole blood metal control levels 1, 2, and 3 (Lyphocheck, Bio-Rad, Hercules, CA, USA) were used for accuracy and precision assurance and quality control purposes.

The limits of detection (LODs) for blood Pb and blood Cd were 3 µg/dL and 0.1 µg/L, respectively. The LOD of urinary Cd was 0.1 µg/L. For urine and blood samples containing Cd and Pb concentrations below the LOD figures, the concentrations assigned were the LOD values divided by the square root of 2 [37].

### 2.4. Normalization of E_Cd_, E_β2M_, and E_NAG_

To correct for interindividual differences in the urine volume and functioning nephrons, urinary excretions of Cd, β_2_M, and NAG (E_Cd_, E_β2M_, and E_NAG_) were normalized to creatinine clearance (C_cr_) using the equation E_x_/C_cr_ = [x]_u_[cr]_p_/[cr]_u_, where x = Cd, β_2_M, or NAG; [x]_u_ = urine concentration of x (mass/volume); [cr]_p_ = plasma creatinine concentration (mg/dL); and [cr]_u_ = urine creatinine concentration (mg/dL). E_x_/C_cr_ was expressed as an amount of x excreted per volume of glomerular filtrate [38]. This C_cr_ normalization is not affected by variations in muscle mass.

For comparison purposes, data from adjusting E_Cd_, E_β2M_, and E_NAG_ to creatinine excretions (E_cr_) were provided. E_x_/E_cr_ = [x]_u_/[cr]_u_, where x = Cd, β_2_M, or NAG; [x]_u_ = urine concentration of x (mass/volume); and [cr]_u_ = urine creatinine concentration (mg/dL). E_x_/E_cr_ was expressed as an amount of x excreted per g of creatinine.

E_cr_ normalization corrects only for differences in urine dilution, and it is affected by a large variation in muscle mass, especially between men and women. E_cr_ adjustment can create non-differential errors/imprecisions that can obscure or even nullify the dose–response relationships [39]. The risk of abnormal E_β2M_ values was not significantly related to E_Cd_ when E_Cd_ and E_β2M_ were adjusted to E_cr_ [40].

### 2.5. Computation of eGFR, Fβ_2_M, TD_β2M_, and FrTD_β2M_

Chronic Kidney Disease Epidemiology Collaboration (CKD-EPI) equations were used to compute eGFR [41]. CKD stages 1, 2, 3, 4, and 5 corresponded to eGFR of 90–119, 60–89, 30–59, 15–29, and <15 mL/min/1.73 m^2^, respectively.

The parameters used to describe kidney handling of β_2_M were (i) the rate of glomerular filtration of β_2_M (Fβ_2_M), mg/d; (ii) the amount of β_2_M undergoing tubular degradation per volume of glomerular filtrate (TD_β2M_/C_cr_), mg/L filtrate; and (iii) the fractional tubular degradation of filtered β_2_M (FrTD_β2M_), decimal fraction. These parameters were computed with the below equations [25].

Fβ_2_M = eGFR[β_2_M]_s_ (1)

TD_β2M_/C_cr_ = [β_2_M]_s_ − E_β2M_/C_cr_ (2)

FrTD_β2M_ = (TD_β2M_/C_cr_)/[β_2_M]_s_ (3)

### 2.6. Analysis for Mediating Effects

**Scheme 1:**
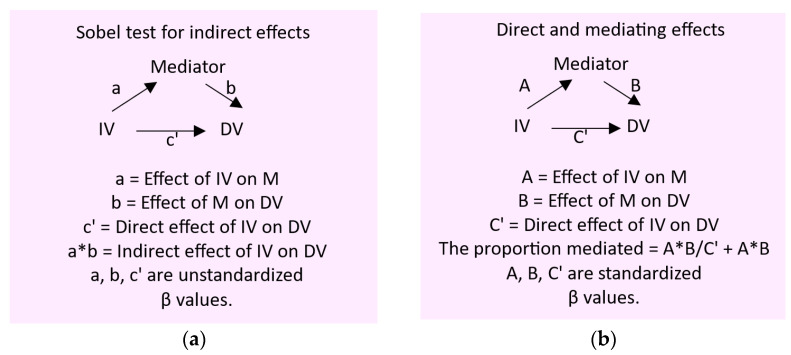
A simple mediation model for cause–effect relationship inference. The unstandardized β coefficients and the Sobel test for the indirect effect of the independent variable (**a**); the standardized β coefficients for the direct and mediating effects of the independent variable (**b**). IV, independent variable; DV, dependent variable.

To gain insight into the mediators of the effects of Cd/Pb exposure on kidney tubular and glomerular functions, we employed the Baron and Kenny method [42,43,44], and their generic simple mediation models are depicted together with statistics parameters (β coefficients) describing the direct and indirect effects of the independent variable tested (Scheme 1).

### 2.7. Statistical Analysis

Data were analyzed with IBM SPSS Statistics 21 (IBM Inc., New York, NY, USA). The Kruskal–Wallis test was employed to assess the variation in any continuous variable across Cd/Pb exposure levels. The Pearson chi-squared test assessed differences in percentages across Cd/Pb exposure levels. Spearman’s rank correlation analysis was used to assess interrelationships among nine variables: FrTD_β2M_, age, BMI, eGFR, FPG, E_Cd_/C_cr_, E_NAG_/C_cr_, and Cd/Pb exposure levels.

The one-sample Kolmogorov–Smirnov test assessed deviation from a normal distribution of any continuous variable. Logarithmic transformation was applied to E_Cd_/C_cr_, E_β2M_/C_cr_, and E_NAG_/C_cr_ that showed rightward skewing before they were subjected to parametric statistics analyses, scatterplots, and linear regressions.

Multiple linear regression modeling was employed to identify the variables contributing to the variability of FrTD_β2M_ among subjects with/without diabetes.

## 3. Results

### 3.1. Cd/Pb Exposure Categorization

The grouping of study subjects was based on the median blood Cd (0.3 µg/L) and the median blood Pb (2.12 µg/dL). Cd/Pb exposure levels 1, 2, and 3 included subjects who had blood Cd and blood Pb levels ≤ medians, blood Cd or blood Pb ≥ the median, and blood Cd and blood Pb both ≥ medians, respectively. The corresponding numbers of subjects in the Cd/Pb exposure levels 1, 2, and 3 were 44, 54, and 39. The characteristics of Cd/Pb categorical exposure groups can be found in Table 1.

Among 137 study subjects, 107 (78%) were women, with an overall mean age (range) of 59.7 (41–80) years. The group with a Cd/Pb exposure level 3 had the highest percentages of smokers (23), diagnosed diabetes (64), hypertension (72), and fasting plasma glucose (FPG) ≥ 126 mg/dL (56).

Nearly half (49%) of study subjects had FPG ≥ 110 mg/dL, commensurate with prediabetes status, and they seemed to distribute equally; the different % distributions of FPG ≥ 110 mg/dL across Cd/Pb exposure groups did not reach a statistically significant level (*p* = 0.115). The overall % of low eGFR was 12.4, and the prevalences of low eGFR across the Cd/Pb exposure levels did not differ (*p* = 0.469).

The parameters showing significant variations across the Cd/Pb exposure levels were [Cd]_b_, [Cd]_u_, [Pb]_b_, E_Cd_, and E_β2M_. The variations in age, BMI, FPG, eGFR, and E_NAG_ across the Cd/Pb exposure levels were not statistically different.

### 3.2. Cd/Pb Exposure and Kidney Handling of β_2_M

**Figure 1:**
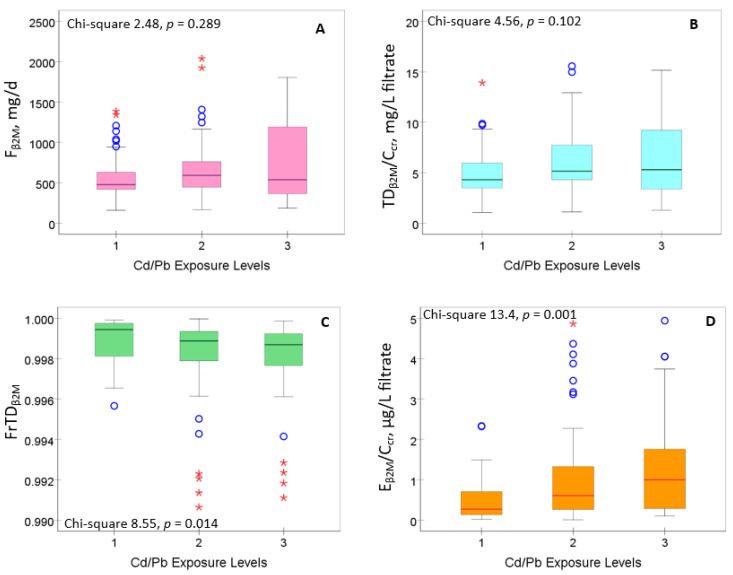
Boxplot distributions of β_2_M homeostasis parameters in subjects grouped by Cd/Pb exposure levels. Boxplots of data on Fβ_2_M (**A**), TD_β2M_/C_cr_ (**B**), FrTD_β2M_ (**C**), and E_β2M_/C_cr_ (**D**) in subjects with Cd/Pb exposure levels 1, 2, and 3, respectively. Each box represents the 25th and 75th percentile values of the variable indicated on the *x*-axis. A horizontal line inside each box represents the median. Circles and asterisks represent outliers.

Figure 1 provides the results of an analysis aimed at identifying the components of β_2_M homeostasis that may be affected by Cd/Pb exposure. The tested β_2_M homeostasis parameters were the filtration of β_2_M (F_β2M_), tubular degradation of β_2_M (TD_β2M_/C_cr_), fractional tubular degradation of β_2_M (FrTD_β2M_), and urinary excretion of β_2_M (E_β2M_/C_cr_). The calculations of these parameters are described in Section 2.5.

Neither F_β2M_ nor TD_β2M_/C_cr_ varied with the Cd/Pb exposure levels (Figure 1A,B). However, FrTD_β2M_ fell as the Cd/Pb exposure levels rose (*p* = 0.014) (Figure 1C), while E_β2M_/C_cr_ rose with the increment of Cd/Pb exposure levels (*p* = 0.001) (Figure 1D). Thus, Cd/Pb exposure appeared to have a significant effect on β_2_M homeostasis, reflected by two parameters, FrTD_β2M_ and E_β2M_/C_cr_.

To gain further mechanistic insights, we conducted a Spearman’s rank correlation analysis to examine the interrelationship of indicators/biomarkers of kidney tubular and glomerular functions (Table 2).

FrTD_β2M_ varied directly with eGFR (*r* = 0.434), and inversely with FPG (*r* = −0.215), E_Cd_/C_cr_ (*r* = −0.527), E_NAG_/C_cr_ (*r* = −0.536), and Cd/Pb exposure (*r* = −0.249). The correlation between FrTD_β2M_ and E_Cd_/C_cr_ was particularly strong, as was the correlation between FrTD_β2M_ and E_NAG_/C_cr_.

The potential tubulo-glomerular effects of Cd/Pb exposure were evident from the eGFR vs. E_NAG_/C_cr_ (*r* = −0.467), E_Cd_/C_cr_ vs. E_NAG_/C_cr_ (*r* = 0.328), and Cd/Pb exposure levels vs. E_Cd_/C_cr_ (*r* = 0.301) correlations.

**Figure 2:**
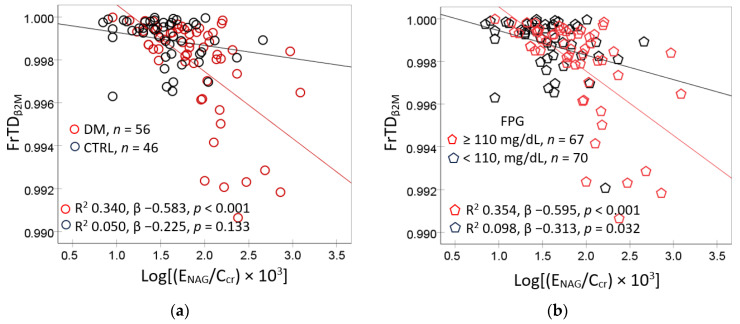
Reduction in fractional tubular degradation of β_2_M as a function of NAG excretion rates. Scatterplots related FrTD_β2M_ to E_NAG_/C_cr_ in subjects with and without diagnosed diabetes (**a**), and those with FPG < 110 and ≥110 mg/dL (**b**). FrTD_β2M_, fractional tubular degradation of β_2_M; NAG, N-acetylglucosaminidase; E_NAG_, urinary excretion of NAG; FPG, fasting plasma glucose concentration; Cd, cadmium; E_Cd_, urinary excretion of Cd.

Scatterplots and regression analyses were used to examine the correlations of FrTD_β2M_ with E_NAG_/C_cr_ in subgroups (Figure 2).

In subjects with diabetes, FrTD_β2M_ rose linearly with E_NAG_/C_cr_ (R^2^ 0.340, β = −0.583, *p* < 0.001), but not in those without diabetes (R^2^ 0.050, β = −0.225, *p* = 0.133) (Figure 2a). In subjects with FPG ≥ 110 mg/dL, FrTD_β2M_ was more closely associated E_NAG_/C_cr_ (R^2^ 0.354, β = −0.595, *p* < 0.001) compared to those who had FPG < 110 mg/dL (R^2^ 0.098, β = −0.313, *p* = 0.032) (Figure 2b).

**Figure 3:**
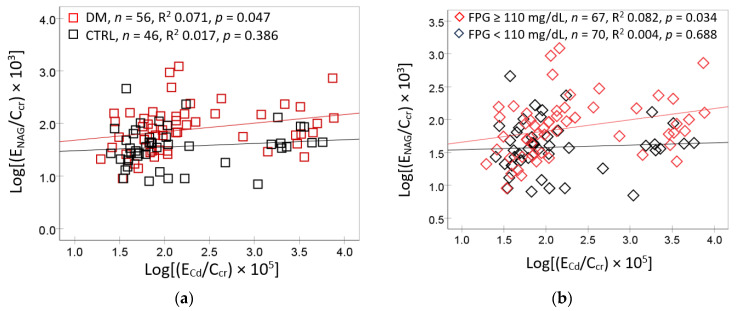
NAG excretion rates as a function of Cd excretion rates. Scatterplots related E_NAG_/C_cr_ to E_Cd_/C_cr_ in subjects with and without diabetes (**a**), and those with FPG < 110 and ≥110 mg/dL (**b**). NAG, N-acetyl-β-D-glucosaminidase; E_NAG_, urinary excretion of NAG; FPG, fasting plasma glucose concentration; Cd, cadmium; E_Cd_, urinary excretion of Cd.

Additional scatterplots and regression analyses were conducted based on the correlation between E_NAG_/C_cr_ and FrTD_β2M_ together with FPG and E_Cd_/C_cr_, identified in Table 2. The results are reported in Figure 3.

In subjects with diabetes, E_NAG_/Ccr rose linearly with E_Cd_/C_cr_ (R^2^ 0.071, *p* = 0.047), but not in the control group (R^2^ 0.017, *p* = 0.386) (Figure 3a). A significant association between E_NAG_/C_cr_ and E_Cd_/C_cr_ was found only in subjects with FPG levels commensurate with prediabetes (R^2^ 0.082, *p* = 0.034) (Figure 3b).

### 3.3. Multiple Linear Regression Models for FrTD_β2M_ in Diabetes vs. Controls

To confirm the results of the FrTD_β2M_ bivariate and regression analyses, a multiple regression model analysis was conducted to adjust for the effects of covariates. The independent variables incorporated in each regression model were for age, BMI, eGFR, E_Cd_/C_cr_, diagnosed diabetes, gender, smoking hypertension, and FPG (Table 3).

All independent variables explained 31.1, 30.1, and 21.5% of the FrTD_β2M_ variability in all subjects (*p* < 0.001), the diagnosed diabetes group (*p* < 0.001), and the controls (*p* = 0.003). FrTD_β2M_ showed a strong positive association with eGFR in both diabetes (β = 0.476, *p* < 0.001) and the controls (β = 0.360, *p* = 0.033).

Intriguingly, an inverse association of FrTD_β2M_ and E_Cd_/C_cr_ was found only in the diabetes group (β = −0.295, *p* = 0.009). A moderate positive association of FrTD_β2M_ and FPG was evident in the control group only (β = 0.247, *p* = 0.033).

### 3.4. Mediating Effects of Cd/Pb Exposure on FrTD_β2M_

**Figure 4:**
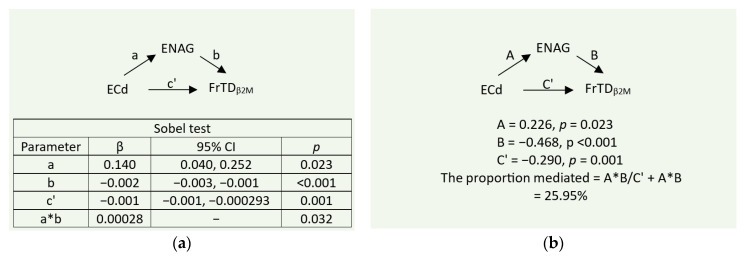
Kidney tubular cell injury as the mediator of Cd effect on β_2_M degradation. A mediating effect of Cd on FrTD_β2M_ by E_NAG_, along with the Sobel test (**a**); the proportion of the effect of Cd mediated by E_NAG_ (**b**). E_NAG_, excretion of NAG; FrTD_β2M_, fractional tubular degradation of β_2_M; CI; bootstrapped confidence interval.

To investigate the mediating role of tubular injury, indicated by E_NAG_/C_cr_, a simple mediation model with a single mediator was employed. As depicted in Figure 4, the independent variable, E_Cd_/C_cr_, was indicative of Cd/Pb exposure, given a significant correlation between E_Cd_/C_cr_ and Cd/Pb exposure levels, revealed in Table 2.

As indicated by the statistical significance of the indirect (a*b) and the direct (c’) effects (Figure 4a), tubular injury (E_NAG_/C_cr_) mediated about 26% of the effect of E_Cd_/C_cr_ on FrTD_β2M_ (Figure 4b).

### 3.5. Mediating Effects of Cd/Pb Exposure on eGFR and FPG

**Figure 5:**
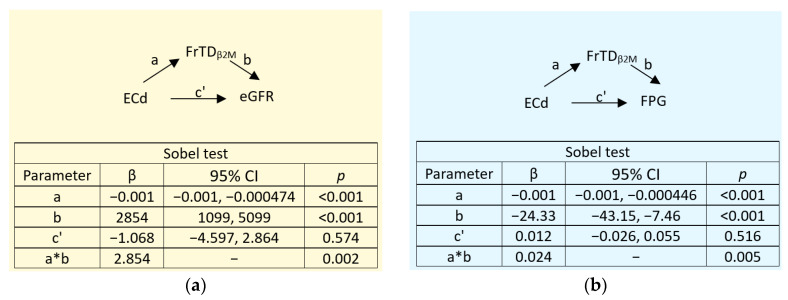
Kidney tubular cell dysfunction as the mediators of Cd effects. The mediating effect of Cd on eGFR (**a**) and FPG (**b**), together with the Sobel test. FrTD_β2M_, fractional tubular degradation of β_2_M; eGFR, estimated glomerular filtration rate; CI; bootstrapped confidence interval; FPG, fasting plasma glucose fractional tubular degradation of β_2_M.

Two additional simple mediation models were conducted with eGFR and FPG as the independent variables to examine the relationships of E_Cd_, FrTD_β2M_, eGFR, and FPG (Figure 5). E_Cd_/C_cr_ was the independent variable that also reflected Pb co-exposure.

As indicated by the statistically significant levels of the indirect effects (a*b) at *p* < 0.001 (Figure 5a) and *p* = 0.005 (Figure 5b), the effects of E_Cd_/C_cr_ on eGFR and FPG both appeared to be mediated fully through a reduced FrTD_β2M_.

## 4. Discussion

The low-level environmental exposure to Cd and Pb experienced by the study subjects was indicated by the median values for blood Pb, blood Cd, and urinary Cd excretion rate (E_Cd_/E_cr_) of 2.12 µg/dL, 0.3 µg/L, and 0.12 µg/g creatinine, respectively. These Cd/Pb exposure levels were less than those found to be associated with an increased risk of diabetes reported in the studies from the U.S. [45,46] and Korea [47]. In the Korean study, a correlation was observed between E_Cd_ and FPG levels, together with the increases in the prevalence of diabetes by 81% and 39% in men and women who had E_Cd_/E_cr_ levels ≥ 2 and ≥3 µg/g creatinine, respectively [47].

As expected from the low E_Cd_ levels and modest sample size (*n* = 137), the correlation between FPG and urinary Cd excretion levels in study subjects (mean 0.98 µg/g creatinine) did not reach statistically significant levels (*r* = 0.166, *p* = 0.053) (Table 2). Notably, however, E_Cd_/C_cr_ showed a positive correlation with a tubular injury biomarker (E_NAG_) (*r* = 0.328, *p* = 0.001). In the subgroup analysis (Figure 3), a significant correlation between E_NAG_ and E_Cd_ was found only in those with diabetes (R^2^ 0.071, *p* = 0.047) and those with FPG ≥ 110 mg/dL (R^2^ 0.082, *p* = 0.034). Thus, an observed tubular injury could be attributed to the Cd/Pb exposure plus the presence of diabetes and/or hyperglycemia (FPG ≥ 110 mg/dL). Hyperglycemia and diabetes are known to have an impact on kidney tubular and glomerular functions [2].

The evidence for the significant impact of co-exposure to low levels of Cd and Pb on the function of tubular cells comes from an analysis of tubular degradation of the filtered protein, β_2_M (Figure 1). The protein β_2_M, by virtue of its small mass, readily passes through the glomerular membrane to the tubular lumen and is reabsorbed and degraded totally by proximal tubular cells [48]. In conventional Cd toxic risk assessment, an increased urinary excretion of β_2_M (E_β2M_/E_cr_) has been used as an indicator of tubular dysfunction. A recent analysis on β_2_M homeostasis, however, has recommended the use of the fractional tubular degradation of β_2_M (FrTD_β2M_) instead of E_β2M_ [25].

Herein, we observed that FrTD_β2M_ dropped as the Cd/Pb exposure levels rose (*p* = 0.014) (Figure 1C). The excretion of β_2_M (E_β2M_/C_cr_) rose with the increment of Cd/Pb exposure levels (*p* = 0.001) (Figure 1D). Thus, Cd/Pb co-exposure even in low doses appeared to have a significant effect on tubular cell function, when FrTD_β2M_ was employed as an effect indicator. Also, we observed that FrTD_β2M_ varied inversely with E_NAG_/C_cr_ in subjects with diabetes (R^2^ = 0.340) (Figure 2a) and those with hyperglycemia (R^2^ = 0.354) (Figure 2b). The results from the mediation analysis (Figure 4) inferred that tubular injury (E_NAG_/C_cr_) mediated about 26% of the reduction in the tubular degradation of β_2_M associated with Cd/Pb exposure.

In the multiple regression model (Table 3), 31% of the variation in FrTD_β2M_ could be accounted for, thereby suggesting that the model with a modest sample size (*n* = 137) was justifiable. The most influential variable was eGFR (β = 0.372), followed by FPG (β = −0.258), diabetes (β = −0.222), and E_Cd_/C_cr_ (β = −0.189). In the subgroup analysis, FrTD_β2M_ was more closely associated with eGFR in subjects with diabetes (β = 0.476) than controls (β = 0.360). In comparison, however, FrTD_β2M_ was inversely associated with E_Cd_/C_cr_ only in the diabetes group (β = −0.295), while it showed a moderate positive association with FPG in the control group only (β = 0.247). These may reflect an increased susceptibility to the nephrotoxicity of low-dose Cd among people with diabetes.

The mediation analysis (Figure 5) revealed the potential connections between reduced FrTD_β2M_, a falling eGFR, and hyperglycemia associated with Cd/Pb exposure. These findings are consistent with studies from Germany and Switzerland. Lower eGFR values were associated with higher blood Pb levels (*r* = 0.318) in a German cohort (*n* = 21,733), where such inverse eGFR/blood Pb associations persisted in a multiple regression analysis, adjusting for covariates of age, gender, FPG, and inflammatory conditions, assessed with circulating C-reactive protein levels [49]. A longitudinal study from Switzerland has causally linked urinary Cd excretion rate to a fall in eGFR at a high rate (≥3 mL/min/1.73 m^2^ per year) [50].

An increased sensitivity to the nephrotoxicity of Cd in people with diabetes was noted in the Cadmibel study [51] and a Swedish cohort study [23]. In empirical studies, histopathological examinations revealed that kidney tubular atrophy and fibrosis were more pronounced in diabetic rats treated with Pb, Cd, manganese, and arsenic compared to the diabetic group without Pb/Cd administration [52,53]. A study on representative Korean adults (*n* = 40,328, aged ≥ 19 years) reported an elevated risk of hypertension in those who had blood Pb ≥ 1.62 µg/dL or blood Cd ≥ 0.67 µg/L. These blood Pb and blood Cd levels are comparable with the participants in the present study [54]. Thus, the relationships between FrTD_β2M_, elevated plasma β_2_M levels, and systolic blood pressure warrant further studies [4,5,6].

The limitations of the present work include a one-time-only assessment of Cd/Pb exposure and outcomes, and an inability to adequately adjust for potential confounders such as alcohol consumption or the use of non-steroidal anti-inflammatory medication. The cross-sectional design limits causal inference, and the modest cohort size reduces statistical power. Longitudinal studies are needed to confirm whether FrTD_β2M_ can serve as an early warning sign of Cd-induced nephrotoxicity. The integration of oxidative stress or inflammatory markers could further support the proposed mechanism.

## 5. Conclusions

This study observed a strong association between the reduced tubular degradation of β_2_M (FrTD_β2M_) and tubular cell injury biomarkers (E_NAG_) in subjects with diabetes (R^2^ = 0.340) and those with FPG levels commensurate with prediabetes (R^2^ = 0.354) who were exposed to Cd and Pb. Through a mediation analysis, tubular injury (E_NAG_) mediated about 26% of the FrTD_β2M_ reduction associated with Cd/Pb exposure. In addition, the mediation analysis revealed the relationships between a reduced FrTD_β2M_, a falling eGFR, and hyperglycemia (FPG ≥ 110 mg/dL) associated with Cd/Pb exposure. The strong association between FrTD_β2M_ and E_NAG_ was observed only in subjects with diabetes and/or hyperglycemia, suggesting an increased sensitivity to the nephrotoxicity of Cd/Pb in people with diabetes.

## Figures and Tables

**Table 1:** Descriptive data on study subjects according to Cd and Pb exposure levels.

Variables	All Subjects *n* = 137	Cd/Pb Exposure Levels			*p*
		Level 1 *n* = 44	Level 2 *n* = 54	Level 3 *n* = 39	
Women, %	78	91	72	72	0.045
Smoking, %	10	0	9	23	0.002
Diagnosed diabetes, %	47	34	46	64	0.023
Hypertension, %	54	51	44	72	0.029
Age (range), years	59.7 (41–80)	59.6 (42–80)	59.8 (45–78)	59.5 (41–78)	0.994
BMI, kg/m^2^	25.6 (4.8)	25.6 (4.2)	25.4 (4.0)	25.9 (6.2)	0.805
FPG_,_ mg/dL	129 (61)	115 (40)	131 (73)	143 (59)	0.099
FPG ≥ 110 mg/dL, %	48.9	38.6	48.1	61.5	0.115
FPG ≥ 126 mg/dL, %	39.4	29.5	35.2	56.4	0.031
eGFR, mL/min/1.73 m^2^	79 (16)	81 (15)	77 (16)	80 (18)	0.461
^a^ Low eGFR, %	12.4	9.1	16.7	10.3	0.469
[β_2_M]_s_, mg/L	5.98 (3.29)	5.01 (2.47)	6.16 (3.12)	6.83 (4.06)	0.099
[Pb]_b_, µg/dL	4.49 (4.78)	2.12 (0.0001)	4.65 (5.63)	6.95 (5.00)	<0.001
[Cd]_b_, µg/L	0.57 (0.70)	0.05 (0.05)	0.60 (0.68)	1.10 (0.73)	<0.001
[Cd]_u_, µg/L	0.65 (1.11)	0.27 (0.69)	0.59 (1.09)	1.17 (1.33)	<0.001
E_cr_-normalized data					
E_Cd_/E_cr_, µg/g creatinine	0.98 (1.86)	0.33 (0.72)	0.99 (2.06)	1.70 (2.20)	0.006
E_β2M_/E_cr_, µg/g creatinine	108 (118)	60 (58)	116 (128)	151 (136)	0.002
E_NAG_/E_cr_, U/g creatinine	10.6 (14.5)	6.67 (4.93)	11.6 (16.7)	13.4 (17.6)	0.288
C_cr_-normalized data					
(E_Cd_/C_cr_) × 100, µg/L filtrate	0.86 (1.68)	0.28 (0.62)	0.86 (1.78)	1.50 (2.11)	0.001
(E_β2M_/C_cr_) × 100, μg/L filtrate	99 (115)	52 (56)	108 (123)	139 (134)	0.001
(E_NAG_/C_cr_) × 100, U/L filtrate	10.2 (17.4)	5.31 (4.03)	10.6 (16.0)	14.6 (24.5)	0.154

eGFR, estimated glomerular filtration rate; ^a^ low eGFR was defined as eGFR values ≤ 60 mL/min/1.73 m^2^; FPG, fasting plasma glucose; β_2_M, β_2_-microglobulin; Cd, cadmium; Pb, lead; [x]_p_, plasma concentration of x; [x]_u_, urine concentration of x; E_cr_, creatinine excretion; C_cr_, creatinine clearance; NAG, N-acetylglucosaminidase. Values for continuous variables are means (standard deviation, SD). Data on NAG were from 102 subjects. All other data were from 137 subjects. The variability of continuous variables across Cd/Pb exposure levels 1, 2, and 3 was assessed with the Kruskal–Wallis test. The distribution of categorial variables was assessed with the chi-square test. For all tests, *p*-values ≤ 0.05 indicate statistical significance.

**Table 2:** Bivariate relationship analysis of the fractional tubular degradation of β_2_-microglobulin.

Variables	Spearman’s Rank Correlation Coefficient						
	FrTD_β2M_	Age	BMI	eGFR	FPG	E_Cd_/C_cr_	E_NAG_/C_cr_
Age	−0.096						
BMI	0.048	−0.262 **					
eGFR	0.434 **	−0.356 **	0.161				
FPG	−0.215 *	−0.222 **	0.184 *	0.089			
E_Cd_/C_cr_	−0.527 **	0.078	−0.083	−0.227 **	0.166		
E_NAG_/C_cr_	−0.536 **	−0.115	0.002	−0.467 **	0.278 **	0.328 **	
Cd/Pb exposure ^a^	−0.249 **	0.009	−0.012	−0.013	0.181 *	0.301 **	0.165

FrTD_β2M_, fractional tubular degradation of β_2_-microglobulin; BMI, body mass index; eGFR, estimated glomerular filtration rate; FPG, fasting plasma glucose concentration. ^a^ Cd/Pb exposure was categorized as Cd/Pb exposure level 1, 2, or 3 using the median blood Cd of 0.3 µg/L and median blood Pb of 2.12 µg/dL. Subjects with blood Cd or blood Pb below median, blood Cd or blood Pb above its median, and blood Cd and blood Pb levels above medians were classed as Cd/Pb exposure levels 1, 2, and 3, respectively. * Significant correlation at the 0.05 level. ** Significant correlation at the 0.01 level.

**Table 3:** Multiple linear regression of FrTD_β2M_ in subjects with and without diabetes.

Independent Variables	FrTD_β2M_					
	All, *n* = 137		Diabetes, *n* = 72		Controls, *n* = 65	
	β	*p*	β	*p*	β	*p*
Age, years	−0.009	0.913	0.095	0.483	−0.079	0.513
BMI, kg/m^2^	0.082	0.300	0.123	0.300	0.082	0.471
eGFR, mL/min/1.73 m^2^	0.372	<0.001	0.476	<0.001	0.360	0.003
(E_Cd_/C_cr_) × 100, µg/L filtrate	−0.189	0.013	−0.295	0.009	−0.102	0.383
Diagnosed diabetes	−0.222	0.021	n/a	n/a	n/a	n/a
Gender	0.057	0.544	0.036	0.790	0.140	0.337
Smoking	−0.016	0.866	0.021	0.877	−0.085	0.559
Hypertension	0.052	0.492	0.172	0.113	−0.239	0.053
FPG, mg/dL	−0.258	0.007	−0.220	0.055	0.247	0.033
Adjusted R^2^	0.311	<0.001	0.301	<0.001	0.215	0.003

FrTD_β2M_, fractional tubular degradation of β_2_M; BMI, body mass index; eGFR, estimated glomerular filtration rate; β, standardized regression coefficient; adjusted R^2^, coefficient of determination; n/a, not applicable. β indicates strength of association of FrTD_β2M_ with nine independent variables (first column). Adjusted R^2^ indicates the proportion of FrTD_β2M_ variability explained by all independent variables. For all tests, *p*-values ≤ 0.05 indicate a statistically significant association of FrTD_β2M_ with an individual independent variable.

## Data Availability

The original contributions presented in the study are included in the article. Further inquiries can be directed to a corresponding author.

## Abbreviations

The following abbreviations are used in this manuscript:

GFR	Glomerular filtration rate
eGFR	Estimated GFR
Cd	Cadmium
E_Cd_	Urinary excretion rate of Cd
Pb	Lead
cr	Creatinine
C_cr_	Creatinine clearance
β_2_M	β_2_-microglobulin
F_β2M_	Rate of glomerular filtration of β_2_M
E_β2M_	Urinary excretion rate of β_2_M
TD_β2M_	Rate of tubular degradation of β_2_M
FrTD_β2M_	Fractional tubular degradation of filtered β_2_M
